# Enhanced microglial dynamics and a paucity of tau seeding in the amyloid plaque microenvironment contribute to cognitive resilience in Alzheimer’s disease

**DOI:** 10.1007/s00401-024-02775-1

**Published:** 2024-08-05

**Authors:** Nur Jury-Garfe, Javier Redding-Ochoa, Yanwen You, Pablo Martínez, Hande Karahan, Enrique Chimal-Juárez, Travis S. Johnson, Jie Zhang, Susan Resnick, Jungsu Kim, Juan C. Troncoso, Cristian A. Lasagna-Reeves

**Affiliations:** 1 Stark Neurosciences Research Institute, Indiana University School of Medicine, Neurosciences Research Building 214G, 320 West 15th Street, Indianapolis, IN 46202 USA; 2 Department of Anatomy, Cell Biology and Physiology, Indiana University School of Medicine, Indianapolis, IN USA; 3 Department of Pathology, Johns Hopkins University School of Medicine, Baltimore, USA; 4 Department of Medical and Molecular Genetics, Indiana University School of Medicine, Indianapolis, USA; 5 Department of Biostatistics and Health Data Science, Indiana University School of Medicine, Indianapolis, USA; 6 Center for Computational Biology and Bioinformatics, Indiana University School of Medicine, Indianapolis, IN USA; 7 Department of Neurology, Johns Hopkins University School of Medicine, Baltimore, USA; 8 Laboratory of Behavioral Neuroscience, National Institute on Aging and National Institute of Health, Baltimore, MD USA

**Keywords:** Alzheimer’s disease, Resilience, Cognitive reserve, Tau, Amyloid plaques, Microglial motility

## Abstract

Asymptomatic Alzheimer’s disease (AsymAD) describes the status of individuals with preserved cognition but identifiable Alzheimer’s disease (AD) brain pathology (i.e., beta-amyloid (Aβ) deposits, neuritic plaques, and neurofibrillary tangles) at autopsy. In this study, we investigated the postmortem brains of a cohort of AsymAD subjects to gain insight into the mechanisms underlying resilience to AD pathology and cognitive decline. Our results showed that AsymAD cases exhibit enrichment in core plaques, decreased filamentous plaque accumulation, and increased plaque-surrounding microglia. Less pathological tau aggregation in dystrophic neurites was found in AsymAD brains than in AD brains, and tau seeding activity was comparable to that in healthy brains. We used spatial transcriptomics to characterize the plaque niche further and revealed autophagy, endocytosis, and phagocytosis as the pathways associated with the genes upregulated in the AsymAD plaque niche. Furthermore, the levels of ARP2 and CAP1, which are actin-based motility proteins that participate in the dynamics of actin filaments to allow cell motility, were increased in the microglia surrounding amyloid plaques in AsymAD cases. Our findings suggest that the amyloid-plaque microenvironment in AsymAD cases is characterized by the presence of microglia with highly efficient actin-based cell motility mechanisms and decreased tau seeding compared with that in AD brains. These two mechanisms can potentially protect against the toxic cascade initiated by Aβ, preserving brain health, and slowing AD pathology progression.

**Supplementary Information:**

The online version contains supplementary material available at 10.1007/s00401-024-02775-1.

## Introduction

Beta-amyloid (Aβ) plaques and neurofibrillary tau tangles (NFTs) have long been known to be causally related to the cognitive manifestations of Alzheimer’s disease (AD) [19]. However, several studies have revealed the existence of aged individuals harboring a high burden of brain lesions at autopsy while remaining cognitively intact, indicating resilience to AD pathology [3, 7, 21, 22, 56, 57, 68, 76, 78, 96]. These individuals have neuritic plaque and NFTs Braak stages at autopsy comparable to those of AD subjects with dementia, and the literature refers to them as resilient [31], Nondemented with AD Neuropathology (NDAN) [83], or asymptomatic AD (AsymAD) [22, 42]. In the present study, we will refer to them as AsymAD. Several reports have provided insight into the resistance of AsymAD individuals to cognitive decline. Specifically, studies have demonstrated that AsymAD brains exhibit no signs of notorious synaptic or neuronal deterioration [8, 42, 73] and, intriguingly, even show larger nuclei and cellular sizes than age-matched healthy controls [79]. Additionally, unlike in the brains of AD subjects with dementia, no phosphorylated tau accumulation occurs within the synapses of AsymAD brains [73, 83]. AsymAD brains have also been found to exhibit a neuroinflammatory profile distinct from that of AD brains, with decreased numbers of activated microglia and astrocyte cells [73], low levels of proinflammatory cytokines, and increased levels of anti-inflammatory cytokines [5]. Advancements in omics and large cohort dataset analyses have enabled the identification of potential cell signatures and molecular mechanisms of resilience, including high processing of energetic pathways involving mitochondrial metabolism and glycolysis, axonal and dendritic growth, and a general increase in protein processing [11, 90, 91].

Despite studies using whole-brain approaches, bulk proteomics, and transcriptomics have helped understand how synaptic preservation and neuron survival occur in AsymAD brains, the molecular mechanisms underlying resilience in the presence of NFTs and amyloid plaques remain poorly understood. Increasing evidence suggests that investigating AD pathology with a spatial approach is important for understanding the molecular pathways involved in neurodegeneration. Moreover, the microenvironment in Aβ plaques plays a crucial role in Aβ-mediated neuroinflammation and tau pathogenesis in AD mouse models [34, 53, 106]. Previous studies have demonstrated that Aβ plaques create a unique environment that facilitates the rapid amplification of proteopathic tau seeds into large tau aggregates, which initially appear in dystrophic neurites surrounding Aβ plaques (NP-tau), followed by the formation and spread of tau aggregates [34]. Moreover, efficient microglial clustering around Aβ plaques mitigates amyloid-driven tau seeding [32]. In the context of AD resilience, one study showed that the area surrounding NFTs in the hippocampus of AsymAD cases exhibits lower levels of proteins associated with inflammation, oxidative stress, and high energy demands than those in AD cases’ hippocampus [100]. Additionally, the brains of AsymAD cases exhibit significant upregulation of phagocytic microglia, which help remove damaged synapses as a protective mechanism [27]. These data suggest that studying the local milieu of AD pathology is crucial for understanding tau-driven synapse damage in the human brain. Nevertheless, further studies are needed to provide detailed insight into the mechanisms underlying Aβ plaque-associated microglial reactivity and tau pathogenesis in the context of resilience to AD pathology.

Herein, using postmortem brain samples from AsymAD cases, AD cases, and age-matched control individuals, we performed a detailed histological characterization of Aβ amyloid plaques and their cellular microenvironment, including microglia and astrocyte activation and tau pathology. We also performed spatial whole-transcriptome analyses to identify and characterize neuroprotective mechanisms operating in the amyloid plaque microenvironment and their potential contribution to cognitive resilience in AsymAD cases. AsymAD brains were more enriched in core plaques than were AD brains, whereas filamentous plaques were predominant in the latter. We also observed strong engagement of microglia around the filamentous plaques in AsymAD brains with a concomitant strong reduction in NP-tau and tau seeding activity compared with that in AD brains. Using spatial whole transcriptomics, we further demonstrated that microglia exhibit significantly upregulated actin-based motility gene expression in the amyloid plaque microenvironment of AsymAD cases. This upregulation potentially heightens membrane dynamics, facilitating efficient migration toward the vicinity of the plaque and promoting the elongation of microglial branches, enhancing their engagement with the plaque. Understanding the local drivers of resilience to AD pathology may provide valuable insights into disease mechanisms and promote the development of interventions to halt neuronal and synaptic damage, thereby preventing the clinical manifestations of AD.

## Materials and methods

### Subjects and clinical-neuropathological classification

We examined middle frontal gyrus (MFG) brain tissue from individuals with histopathological findings of AD (i.e., amyloid plaques, hyperphosphorylated tau and NFTs) and healthy aged-matched controls from the Johns Hopkins Alzheimer’s Disease Research Center (ADRC) and the Baltimore Longitudinal Study of Aging (BLSA). The cognitive status preceding death was obtained from detailed neuropsychological assessments reports, including the Mini Mental State Examination (MMSE) [25] and Clinical Dementia Rating (CDR) [63]. The diagnosis of dementia made according to the criteria outlined in the Diagnostic and Statistical Manual of Mental Disorders [2], and guidelines established by the National Institute of Neurological and Communication Disorders and Stroke-Alzheimer’s Disease and Related Disorders [62]. Expert consultation during clinical conferences enriched the diagnostic process. The Johns Hopkins Brain Resource Center (BRC) provided the cognitive status and neuropathologic data. All subjects received their last clinical evaluation within 15 months before death. The postmortem brains were classified into aged-matched controls individuals, AsymAD cases, and AD cases as described in previously clinical and neuropathological studies [41–43, 79].

### Tissue processing and neuropathologic evaluations

All brains were examined in the Division of Neuropathology at Johns Hopkins University. The brains were weight and hemisected through the midline, and the right cerebral hemisphere was cut serially into 1-cm coronal slabs. For diagnostic purposes, tissue blocks were fixed in 10% neutral buffered formalin, processed overnight, and embedded in paraffin. The tissue blocks were cut at 5 μm and stained with hematoxylin and eosin. Selected sections were treated with H_2_O_2_ and blocked with 3% normal goat serum in Tris-buffered saline for immunostaining with antibodies against phospho-tau (Ser202, Thr205) AT8 (1:200, MN1020, Invitrogen), Aβ-amyloid (1:500, clone 6E10, 803023, BioLegend), α-synuclein (1:500, clone 42, 610787, BD), anti-TDP-43 (phosphorylation independent) (1:1000, rabbit polyclonal, 12782-1-AP, Proteintech) and anti-TDP-43 phosphorylation-dependent at serine 409/410 (1:200, clone 1D3, 829901, BioLegend). For the evaluation of AD pathology, the Hirano-Silver staining method [105] and AT8 immunostaining were used to stage the neuritic plaque density according the Consortium to Establish a Registry for Alzheimer’s Disease (CERAD) criteria [80]. Hyperphosphorylated tau pathology was determined using the Braak staging method [1, 9, 10]. Tau pathology progression in cases with Braak stages of V/VI was assessed by identifying hyperphosphorylated tau lesions in the visual cortex (Brodmann’s areas 17 & 18) (Supplementary Fig. 1). Aβ-amyloid distribution was assessed using an anti-Aβ-amyloid antibody according to the Thal phase system [93]. AD neuropathologic changes were staged according to the NIA-AA criteria [40]. Assessment of TDP-43 changes in AD cases was conducted as recommended by Nelson et al. 2019 and 2023 [65, 66]. Alpha-synuclein lesions were evaluated according to the methods of McKeith et al. [61] and primary age-related tauopathy (PART) as described in Crary et al. [16]. AD and AsymAD cases were selected according to age (mean [SD], 82.68 [10.4] years in AD cases and 88.15 [5.5] years in AsymAD cases), NFTs Braak stages (5.32 [0.82] in AD cases and 4.62 [0.76] in AsymAD cases), Thal stage (4.72 [0.75] in AD cases and 4.25[0.45] in AsymAD cases) and TDP43/LATE status (0.94 [0.94] in AD cases and 0.54 [0.82] in AsymAD cases). None of the control individuals or AD or AsymAD cases exhibited Lewy bodies in the examined sections. Chronic traumatic encephalopathy (CTE) was not reported for any of these cases. The neuropathological and clinical details for each case are provided in Table 1.

**Table 1:** Clinical and neuropathological characteristics of the cases included in the study

Number	FDX	CDX	Age	Sex	Braak Stage	CERAD	Thal	ADNC level	TDP43/LATE	CDR	MMSE	APOE	RACE	PMD
1	Control	Normal	68	M	II	0	0	No	0	0	27	3/3	W	10
2	Control	Normal	89	F	II	0	0	No	0	0.5	30	3/3	W	4
3	Control	Normal	52	F	III	0	0	No	0	NA	NA	3/3	W	14
4	Control	Normal	77	M	0	0	2	No	NA	NA	NA	NA	W	13
5	Control	Normal	68	F	II	0	2	No	0	0	NA	NA	W	12
6	Control	Normal	72	M	II	0	0	No	0	0	29	3/3	W	10
7	Control	Normal	66	M	II	0	0	No	NA	NA	NA	NA	W	10
8	Control	Normal	88	F	II	0	0	No	0	NA	NA	NA	W	9
9	Control	Normal	95	M	III	0	0	No	2	0	NA	2/3	W	17
10	Control	Normal	71	F	II	0	1	No	0	0.5	28	4/4	W	16
11	Control	Normal	94	M	III	0	1	No	1	0	30	3/4	W	16
12	Control	Normal	79	F	II	0	1	NA	NA	NA	NA	NA	W	33
13	Control	Normal	79	M	II	A	1	Low	0	0	NA	2/4	W	16
14	AD	Demented	86	F	IV	B	NA	Intermediate	2	3	19	3/4	W	5
15	AD	Demented	89	M	IV	B	5	Intermediate	2	2	15	3/4	W	8
16	AD	Demented	89	M	IV	B	5	Intermediate	2	2	15	3/4	W	8
17	AD	Demented	97	F	IV	B	2	NA	NA	NA	NA	NA	W	11
18	AD	Demented	86	M	V	C	5	High	0	2	NA	2/3	W	5
19*	AD	Demented	75	M	V	C	5	High	1	NA	17	NA	W	13
20	AD	Demented	73	M	V	C	5	High	0	NA	23	4/4	W	7
21	AD	Demented	96	M	V	B	5	High	0	3	11	3/3	W	NA
22*	AD	Demented	88	F	V	C	5	High	2	NA	NA	NA	W	11.5
23	AD	Demented	80	F	VI	B	5	High	2	NA	22	NA	W	2
24	AD	Demented	76	M	VI	C	5	High	1	NA	NA	NA	W	15
25	AD	Demented	88	F	VI	C	4	High	0	0	14	NA	W	8
26	AD	Demented	87	F	VI	C	4	High	0	3	NA	NA	W	6.5
27	AD	Demented	55	F	VI	C	5	High	0	NA	7	NA	W	15
28	AD	Demented	78	F	VI	C	5	High	0	NA	1	NA	W	8
29	AD	Demented	85	F	VI	C	5	High	2	3	5	3/4	W	5
30	AD	Demented	65	M	VI	C	5	High	1	NA	NA	NA	W	11
31	AD	Demented	89	F	VI	C	5	High	0	NA	NA	NA	W	7
32	AD	Demented	89	M	VI	C	5	High	2	NA	NA	NA	W	9.5
33	AsymAD	Normal	92	F	IV	B	4	Intermediate	NA	0	29	3/3	W	12
34	AsymAD	Normal	91	M	IV	C	NA	Intermediate	0	0	30	2/3	W	16
35	AsymAD	Normal	75	M	IV	C	4	Intermediate	0	0	29	3/4	W	24
36	AsymAD	Normal	94	M	IV	C	4	Intermediate	2	0	28	3/3	W	14
37	AsymAD	Normal	92	F	IV	C	4	Intermediate	1	0	30	3/3	W	18
38	AsymAD	Normal	92	F	IV	B	4	Intermediate	NA	0	29	3/3	W	12
39	AsymAD	Normal	92	M	IV	B	4	Intermediate	0	0	NA	3/3	W	8.5
40	AsymAD	Normal	92	F	V	C	5	High	2	0	28	3/3	W	13.5
41	AsymAD	Normal	85	F	V	B	5	High	0	0.5	30	3/3	W	27
42*	AsymAD	Normal	89	F	V	C	4	High	0	0	30	2/3	W	3
43	AsymAD	Normal	85	F	V	C	4	High	1	0	NA	3/3	A	9
44	AsymAD	Normal	81	M	VI	C	5	High	0	0.5	28	3/4	NA	16
45	AsymAD	Normal	86	M	VI	B	4	High	0	NA	NA	NA	W	8

*FDX*: final diagnosis, *CDX*: clinical diagnosis, *ADNC*: Alzheimer’s disease neuropathologic change, *TDP43/LATE*: limbic-predominant age-related TDP-43 encephalopathy, CDR: clinical dementia rating, *MMSE*: mini-mental state examination, *PMD*: post-mortem delay*, W*: white, *A*: African American, *N.A.*: Not available

*Cases used for GeoMx analyses

### Immunofluorescence in postmortem human tissue

Formalin-fixed, paraffin-embedded (FFPE) 5-μm MFG sections were deparaffinized in xylene, rehydrated in an ethanol gradient (100–30%), and washed with deionized water. Then, the sections were heated to 95 °C in 1X EDTA buffer, pH 8.5, antigen retrieval solution (E1161, Sigma Aldrich) for 20 min in a thermoregulated bath. For ARP2 and CAP1, an additional 15 min step of incubation with 50% methanol and a subsequent 1 min incubation with proteinase K (1 mg/ml) before the antigen retrieval incubation, were performed. After washing twice with TBS for 5 min, the sections were blocked with Animal-free Blocker® and a diluent solution (SP-5035, Vector Laboratories) supplemented with 0.1% Triton X-100 for 1 h. at RT. The sections were incubated overnight at 4 °C with primary antibodies (phospho-tau (Ser202, Thr205) AT8 (1:100, MN1020, Invitrogen), Aβ-amyloid (6E10) (1:200, MBS488263, MyBiosource), β-Amyloid 17–24 (4G8) (1:200, SIG-39220, Covance), anti-IBA1 (1:300, 019-19741, Wako), anti-GFAP (1:300, ab1218, Abcam), anti-ARP2 (E-12) (1:100, sc-166103, Santa Cruz) and anti-CAP1 (1:200, 68207-1, ProteinTech). The next day, the sections were quickly washed three times in TBS and incubated for 2 h. with a 1:500 ratio of Alexa Fluor secondary antibodies: goat anti-rabbit Alexa Fluor 488 (A11008, Invitrogen), goat anti-mouse Alexa Fluor 488 (A32723, Invitrogen), donkey anti-mouse CF647 (SAB4600176, Sigma) and donkey anti-rabbit Alexa Fluor 568 (AB175470, Abcam), diluted in blocking solution followed by three washes with TBS (5 min each). Double immune detection was employed for ARP2 and CAP1 immunoassays utilizing goat anti-mouse 488 to enhance the antibody signal, followed by a 1 h. incubation with donkey anti-goat 488 (AB150129, Abcam). Amyloid structures were stained with 1% thioflavin S (diluted in 50% TBS/ethanol) or NucBlue (1:100, R37605, Invitrogen) for 20 min at RT followed by five 5 min washes with TBS. Finally, the sections were incubated with TrueBlack (23,007, Biotium) for 30 s. and then washed three times with TBS.

### Image analysis

Brightfield images were acquired using a BH-2 Olympus microscope. Fluorescence imaging was performed using a Nikon A1-R laser scanning confocal microscope coupled with Nikon AR software v.5.21.03.1 Postprocessing and analyses were conducted using ImageJ (National Institutes of Health, v1.53c). Phospho-tau quantification in neurites using brightfield images was conducted as previously described [14]. For plaque classification analyses, 15–20 μm z-stacks were imaged at 60× magnification, and a circularity analysis of thioflavin S plaques was performed using the ‘‘Shape Descriptors’’ plugin in ImageJ. Cut-offs for plaque circularity were defined as previously described [77, 106], where filamentous plaques had a circularity score of 0.00–0.14 and compact plaques had circularities exceeding score of 0.30. Plaques with a circularity score range of 0.15–0.28 were classified as displaying intermediate phenotypes. AT8, IBA1, and GFAP coverage around plaques was quantified as previously described [64] with some modifications; regions-of-interest (ROIs) were traced along 50 μm of plaque perimeter across serial images. Defined ROIs were applied to the AT8/IBA1/GFAP channels, and the percentage of immunoreactivity/signal intensity in the area within the ROI was quantified. Twenty to thirty plaques were quantified per sample.

For the quantification of ARP2 and CAP1, ROIs were traced along 50 μm of the plaque core, and the immunoreactivity/signal intensity was measured within the selected area. IBA1 mask selection was applied to quantify the selected signals exclusively within IBA1-positive areas. Image fields farther than 100 μm from the amyloid plaques were considered “plaque-free areas”.

### Western blot analysis

Postmortem MFG tissues were homogenized in TBS buffer at a ratio of 1:10 (wt/vol) with Pierce Protease and Phosphatase Inhibitor Cocktail (A32965, ThermoScientific) on ice. The tissue lysate was sonicated and centrifuged at 15,000×*g* for 15 min at 4 °C. Protein concentrations were measured using a BCA protein assay kit (Bio-Rad Laboratories, Inc.). Electrophoresis was performed using 20 μg of protein lysates, which were resolved in a 4–12% SDS–PAGE gel (CriterionTM TGXTM, Bio-Rad Laboratories, Inc.) and transferred to a nitrocellulose membrane (Immobilon®-P, Millipore) that was blocked with EveryBlot blocking buffer (12010020, Bio-Rad), followed by overnight incubation with the following primary antibodies diluted in 5% BSA TBS 0.1% Tween: HT7 (1:300, MN1000, Thermo Fisher), phospho-tau (Ser202, Thr205) AT8 (1:1000, MN1020, Invitrogen), PHF1 (1:1000, Peter Davies Antibodies) and MC1 (1:1000, Peter Davies Antibodies). Horseradish peroxidase (HRP) secondary antibodies (goat anti-mouse HRP conjugated (1:10,000, 626820, Invitrogen) were added, and the membranes were incubated for 2 h. at RT. The proteins were detected with Supersignal West Pico (34580, Thermo Scientific). Imaging was conducted using an iBright 1500 (Invitrogen) imager, and the data were analyzed with ImageJ Fiji. Nine cases per group were included in the western blot analyses, comprising three AD and AsymAD cases each with NFTs Braak stages of IV, V and VI.

### Size exclusion chromatography (SEC)

SEC was performed as previously described [60]. Briefly, the column was equilibrated, and the samples were clarified by centrifugation at 10,000×*g* for 10 min. The protein concentrations of the frozen samples were quantified by BCA assay, and 1–5 mg of total protein per supernatant was removed for separation. The supernatant was concentrated with a 0.5 ml 3 K Amicon centrifugal filter (UFC5003, Millipore Sigma) to ~ 200 µl and loaded onto the column via sample loop injection. One ml fractions were collected into tubes containing EDTA-free protease inhibitor (11873580001, Roche) at a flow rate of 0.3 ml min^−1^. Four cases per group were included in the SEC analyses, comprising two AD and AsymAD cases each with NFTs Braak stages of V and VI.

### Human tau ELISA

An enzyme-linked immunosorbent assay (ELISA) was performed on total and SEC fractions using the Tau (Total) Human ELISA Kit (KHB0041, Invitrogen) following the manufacturer’s directions. Lysates were diluted 1:50,000 in blocking buffer; F7–F14 at a ratio of 1:2000 and F15–F22 at a ratio of 1:20,000.

### Tau-seeding assay

The seeding assay was performed as previously described [36, 60]. Briefly, TauRD P301S FRET biosensor cells (CRL-3275, American Type Culture Collection (ATCC)) were plated at 35,000 cells per well in 130 µl of medium in a 96-well plate and incubated at 37 °C overnight. The next day, the cells were transfected with total protein lysates and SEC fractions from control, AD, and AsymAD MFGs (20 µg total protein per well). After harvesting the cells, flow cytometry was conducted with a BD LSR Fortessa X-20 with a high-throughput sampler using BD FACS Diva v8.0 software. FlowJo v10.0 was used for the data analysis. Seeding was quantified by integrated FRET density, defined as the product of the percentage of FRET-positive cells and the median fluorescence intensity of FRET-positive cells.

### NanoString GeoMx™ human whole transcriptome atlas (WTA)

Slide preparation was performed following the GeoMx WTA kit manufacturer’s instructions. Briefly, FFPE MFG sections from AsymAD and AD brains (highlighted with asterisks in Table 1) were deparaffinized and rehydrated followed by antigen and target retrieval and in situ hybridization at 37 °C for 16–24 h. The next day, the slides were washed and incubated for 1 h. with the following morphology markers: β-Amyloid (D54D2) Alexa Fluor 594 conjugated (1:100, Cell Signaling, 35363) and the nuclear marker Syto 13 (1:50, NanoString, 121301310). Slides were loaded into the GeoMx Digital Spatial Profiler (DSP) instrument and scanned to capture fluorescence images used to select ROIs. A 60–100 μm diameter circle was selected surrounding each amyloid plaque. Sixteen to eighteen gray matter ROIs per condition were selected. UV-cleaved oligonucleotides from each spatially resolved ROI were aspirated and collected in a 96-well collection plate for library preparation with Seq Code primers and sequenced on an NextSeq500 sequencer instrument (Illumina). Digital count conversion (DCC) files were obtained using the Illumina DRAGEN Sever v4. Then, DCC files were transferred to the GeoMx DSP Analysis suite v.3.0.0.109 for data quality control (QC) and normalization. The analysis pipeline was conducted using the GeoMx DSP user manual (MAN-10154-01). Differential gene expression (DEG) analyses (*p* < 0.05 with multiple testing correction, fold-change > 1.5) and pathway enrichment analyses were performed using the DEGs found in AsymAD cases. Cell deconvolution and cell abundance analysis were performed using the NanoString SpatialDecon R library [17]. To run the R library package, cell profile matrices derived from previous datasets were built [47, 59, 69, 88]. The cells were clustered into 13 different clusters for the profile matrix using the K-nearest neighbor (KNN) algorithm of Seurat with a resolution of 0.5 [86]

### Manipulation of ACTR2 in a human microglia cell line and scratch assay

The human microglial cell line (HMC3) was cultured in a 96 well-plate as previously described [46]. Twenty nanograms per well were used to transfect plasmids containing human ACTR2 cDNA (VB900143-9176fnc, VectorBuilder) and ACTR2-targeting shRNA (VB231025-1394uat, VectorBuilder) with Lipofectamine™ 3000 (Invitrogen) following the manufacturer’s instructions. An empty vector (VB190731-1138syk, VectorBuilder) and a scrambled RNA (VB010000-9489hhg, VectorBuilder) sequence were used as controls for overexpression and downregulation, respectively. Three days post transfection, ACTR2 RNA was analyzed by qRT-PCR using TaqMan probes (Assay ID: Hs00855199-g1, ThermoFisher). The human GAPDH endogenous control was used as a normalization reference. Twenty-four hours after transfection, a scratch was made in the middle of the wells using the Incucyte® 96-well scratch woundmaker tool (Cat. No. 4563, Sartorius), and the cells were imaged for 72 h with a Leica THUNDER imager system. ImageJ Fiji and the Wound Healing Size plugin were used to calculate the percentage of scratch area, as previously described [87].

### Statistical analyses

All the statistical analyses and graph designs were performed using GraphPad Prism v9.5.0 (525). The data were first analyzed for normality (Shapiro–Wilk test) followed by statistical tests. The results in column graphs represent the mean ± SEM. For histology, immunofluorescence, and biochemical experiments, the Student’s *t*-test was used to compare between two groups. One-way ANOVA followed by multiple comparisons was employed for comparisons of three groups, and two-way ANOVA was used to analyze two variables simultaneously. For all tests, a *p *value of 0.05 was considered to indicate significance. For GeoMx HuWTA analyses, the *p* values were adjusted for multiple analyses using the Benjamini–Hochberg procedure with a false discovery rate (FDR) of 0.01. Data collection and analyses were performed by a researcher blinded to the experimental conditions.

## Results

### Morphologically distinct proportion of amyloid plaques and attenuated tau pathology surrounding amyloid plaques in AsymAD and AD brains

**Fig. 1:**
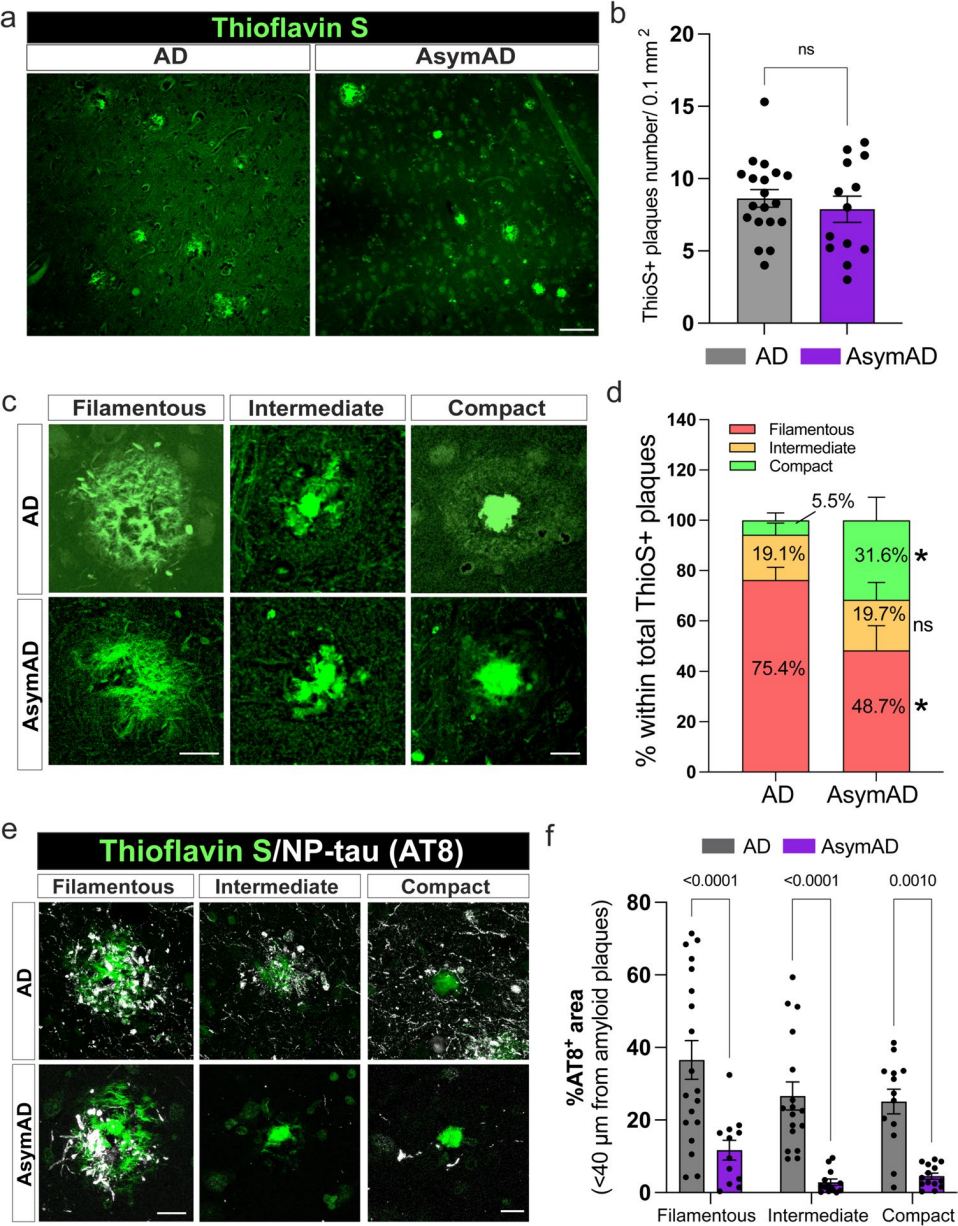
Thioflavin S plaque characterization and the accumulation of tau aggregates in dystrophic neurites (NP-tau) in AsymAD versus AD brains. **a** Thioflavin S staining of the middle frontal gyrus (MFG) of AD and AsymAD brains. Scale bar: 40 μm. **b** Quantification of the total number of thioflavin S plaques. The data are shown as the mean ± SEM, unpaired Student’s *t* test; *n* = 13–19 cases per group. **c** Representative morphologies of each plaque type classified based on the circularity score. Scale bar: 20 and 10 μm **d** Proportions of filamentous, intermediate, and compact thioflavin S plaques were quantified. **e** Confocal images of NP-Tau (AT8; white) and the three previously classified thioflavin S amyloid plaque phenotypes (green) in AD and AsymAD MFGs. Scale bar: 20 and 10 μm **f** Percentage of area positive for AT8 staining surrounding amyloid plaques 50 μm in diameter. *n* = *13–19* cases per group. In d and f, the data are shown as the means ± SEMs, 2-way ANOVA, and Šídák’s multiple comparisons test (in D, *p* value *; 0.016 and *; 0.028)

Given that autopsies of AsymAD and AD brains revealed comparable amyloid plaque and NFTs burdens **(**Supplementary Fig. 2), we investigated whether there were any differences in plaque morphology between these presentations. To do so, we evaluated the amyloid plaque phenotype in the MFG of thioflavin S-stained sections (Fig. 1a). Our analysis of the total number of amyloid plaques within the MFG did not reveal significant differences between AD and AsymAD cases (Fig. 1b). Using previously described plaque classifications [77, 106] and circularity analyses to distinguish compact plaques from plaques with a filamentous or an intermediate morphology (Fig. 1c), we observed that compared with AD cases, AsymAD cases exhibited a significant reduction in the proportion of filamentous plaques, with a concomitant increase in compact plaques (Fig. 1d). Using the 6E10 and 4G8 anti-Aβ-amyloid antibodies, we confirmed that the three previously classified amyloid plaques were positive for Aβ-amyloid (Supplementary Fig. 3). Interestingly, it was previously reported that filamentous plaques are neurotoxic whereas compact dense-core plaques are relatively benign [38, 52]. In the context of AD or aging, the formation of filamentous plaques has been associated with the phosphorylation of tau within dystrophic neuritis [13, 53, 106], which subsequently contributes to the formation of neuritic plaques (a subset of Aβ plaques surrounded by and containing dystrophic neurites) during AD progression [97]. Moreover, amyloid-plaques create a unique molecular environment that facilitates the seeding and spreading of tau pathology. These highly phosphorylated tau species in dystrophic neurites surrounding Aβ plaques (NP-Tau) aggregate faster and spread more widely than tau in NFTs [34]. To investigate whether a reduction in filamentous plaques could impact the formation of NP-tau in the plaque niche of AsymAD MFGs, we performed double-immunofluorescence using thioflavin-S and an AT8 antibody, which recognizes the phospho sites Ser202 and Thr205 within the tau protein (Fig. 1e). Our findings revealed that in the AsymAD MFGs, regardless of the type of plaque, there was a dramatic decrease in NP-tau within the plaque microenvironment in comparison with that in the AD MFGs (Fig. 1f), indicating that although both AsymAD and AD cases exhibit neuritic plaques, the dystrophic neuritic component induced by tau is notably more pronounced in AD cases. Interestingly, in comparison to those in AD MFGs, AsymAD MFGs did not exhibit significant differences in NFTs (Supplementary Fig. 4a, b). However, there was a noticeable reduction in AT8-positive neuritic staining in the MFG area (Supplementary Fig. 4c).

### The seeding capacity and biochemical characterization *of tau* in AsymAD cases revealed a lack of pathological tau features

**Fig. 2:**
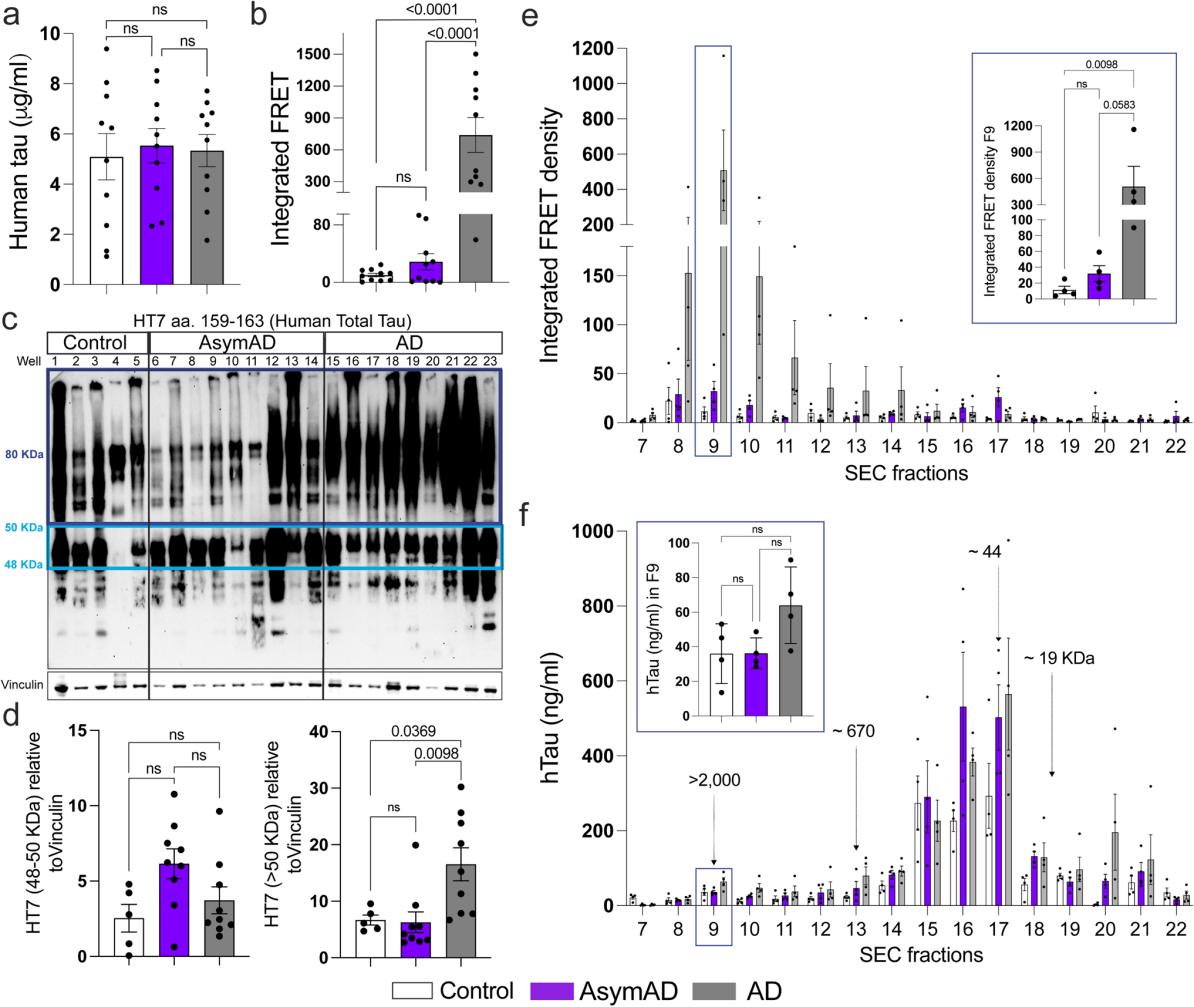
**Tau **seeding activity and biochemical characterization of Tau in AsymAD, AD and age-matched control brains**. a** Total tau detected by ELISA in MFG protein fractions from control, AsymAD, and AD brains **b** Tau seeding activity of total protein fractions. **c, d** Western blot of total HT7 and quantifications of the monomer band (between 40 and 50 kDa, light blue) and the oligomer bands (above 80 kDa, dark blue) (d). The data are shown as the mean ± SEM*.* Experiments were performed with *n* = 10 (ELISA and tau seeding activity) or *n* = 5–9 samples (Western blot) per group. **e** Tau seeding activity of size exclusion chromatography (SEC) fractions. **f** Total tau detected by ELISA in SEC fractions from MFG brain lysates. In e and f, the inset shows the seeding activity (**e**) and tau concentration (**f**) of SEC fraction 9 (F9) containing high-molecular-weight tau (> 2000 kDa). The data are shown as the mean ± SEM*.* Significance was determined by one-way ANOVA, and experiments were performed with *n* = *4* cases per group

Given the reduced levels of NP-tau surrounding the plaque microenvironment in AsymAD cases, we next investigated whether this phenomenon influences soluble tau pathology. First, we evaluated the tau-seeding activity of TBS-soluble lysates from control, AsymAD, and AD MFGs by transfection into tau RD P301S fluorescence resonance energy transfer (FRET) biosensor cells and quantified the integrated FRET density by flow cytometry as previously described [60, 71]. Although total tau levels among age-matched controls and AsymAD and AD MFGs were similar (Fig. 2a), tau in AsymAD brain lysates produced low levels of seeding activity, unlike that in AD lysates (Fig. 2b). Several groups have demonstrated that tau oligomers, which form prior to and independent of NFTs, are the toxic agents responsible for synaptic dysfunction in AD and drive cognitive decline [26, 49, 50, 58, 95]. To better understand our previous findings, we conducted a biochemical characterization of tau by immunoblotting. Our results indicate that AsymAD brain lysates exhibit significantly lower levels of oligomeric tau species than those observed in AD brain lysates (Fig. 2c, d). Furthermore, tau species in the in AsymAD TBS-soluble fraction demonstrated reduced in the pathological phospho-epitopes PHF1 and AT8 (Supplementary Fig. 5a–d). We also observed an increase in misfolded tau (MC1) levels between control and AD cases, whereas no differences were detected between AsymAD and control individuals (Supplementary Fig. 5e, f). Overall, these data indicate diminished pathological tau features in AsymAD MFGs. Our laboratory has recently demonstrated that the primary source of tau seeding activity in AD cases, corresponds to soluble high molecular weight (HMW) tau species; thus, HMW tau-containing particles are one of the predominant toxic entities [60]. We performed size exclusion chromatography (SEC) on TBS-soluble MFG lysates to gain additional insights into the size distribution of tau-seeding species in AsymAD cases. As we previously reported [60], the tau species with the strongest seeding activity in AD cases was a HMW tau in fraction 9 (> 2000 kDa) (Fig. 2e), representing a small percentage of the total tau in the brain (Fig. 2f). Interestingly, although the levels of total tau in fraction 9 were similar in the AD, AsymAD, and control samples (Fig. 2f, inset), tau in the AsymAD group showed a significant decrease in seeding activity to that in the AD group (Fig. 2e, inset). These results suggest that biochemically, the soluble tau present in AsymAD MFGs is more likely to resemble that in age-matched healthy control brains than in AD brains.

### Increased microglia surrounding filamentous amyloid plaques in AsymAD cases

**Fig. 3:**
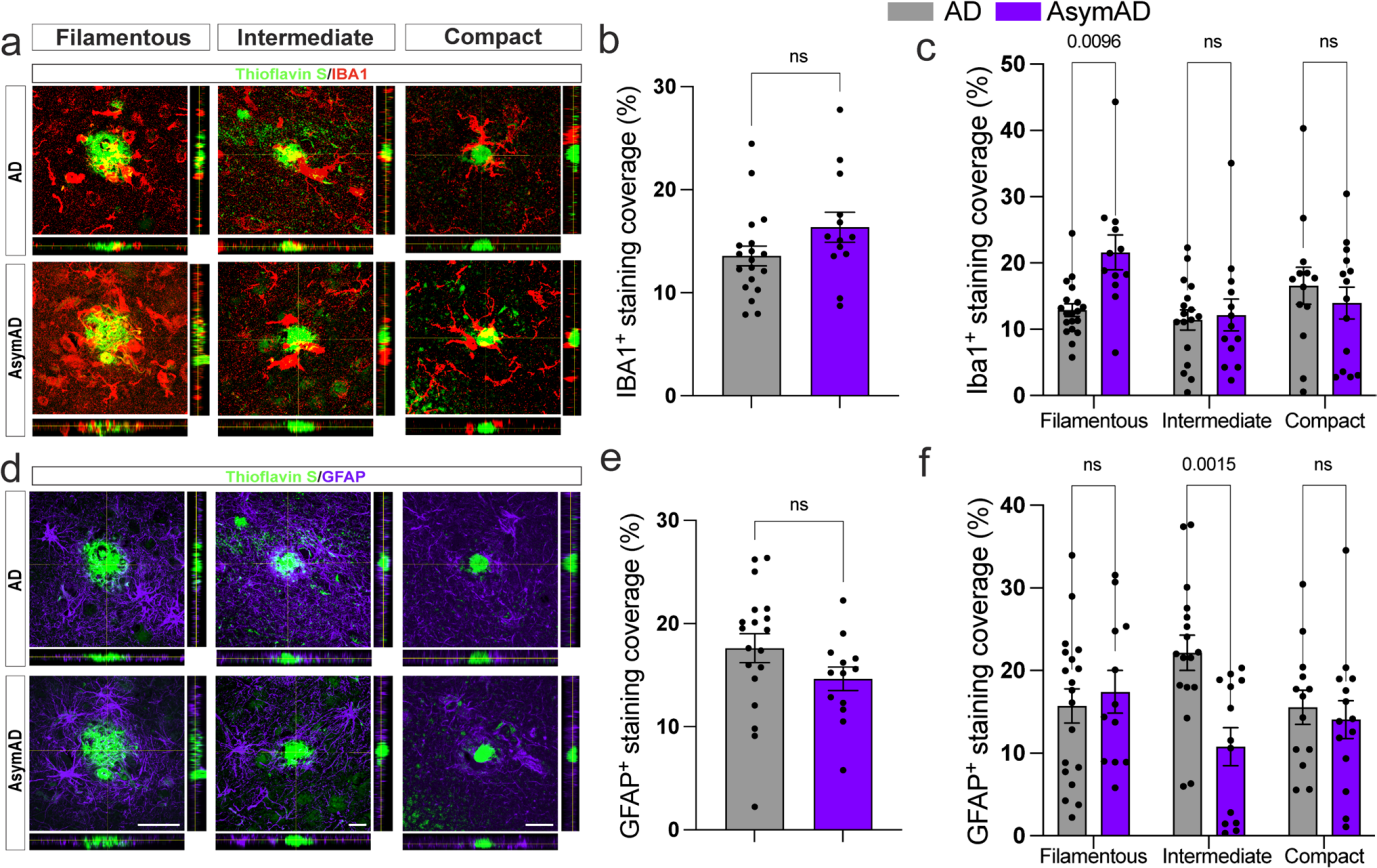
Microglia and astrocytic coverage around compact, intermediate, and filamentous amyloid plaques between AD and AsymAD cases. Staining for the activated microglial marker IBA1 (red) (**a**) and the activated astrocytic marker GFAP (purple) (**d**) around the three thioflavin S amyloid plaque phenotypes. Scale bars: 50, 15, and 20 μm for filamentous, intermediate, and compact images, respectively. Quantification of overall IBA1 (**b**) and GFAP (**e**) staining. The data are shown as the means ± SEMs; unpaired Student’s *t* test; *n* = 14–19 cases per group. IBA1 (**c**) and GFAP (**f**) coverage per plaque phenotype. The data are shown as the means ± SEMs; 2-way ANOVA followed by Šídák’s multiple comparisons test; *n* = 13–19 cases per group

Microglial and astrocytic interactions with Aβ amyloid plaques have been associated with amyloid plaque development and neuritic damage [81]. In this context, microglia appear crucial to the initial appearance and structure of plaques and, following plaque formation, promote a chronic inflammatory state modulating neuronal gene expression changes in response to Aβ during AD pathology [84]. Furthermore, microglia limit diffuse plaques by constructing and maintaining dense compact-like plaque properties in AD mouse models and humans, thereby blocking the progression of neuritic dystrophy and reducing tau phosphorylation in the local plaque environment [12, 38, 106]. To investigate whether the differential proportion of amyloid plaques and decreased NP-tau accumulation found in AsymAD versus AD brains correlate with the dysregulation of plaque-associated microglial and astrocytic responses, we performed immunofluorescence analysis with anti-IBA1 and anti-GFAP to visualize activated microglia and astrocytes associated with Aβ plaques, respectively (Fig. 3a, d). A trend toward increased overall IBA1 and decreased overall GFAP coverage in AsymAD cases was observed, although the difference was not significant (Fig. 3b, e). However, when we measured the percentage of IBA1- and GFAP-positive staining surrounding the three previously classified plaque types, we detected significantly greater levels of IBA1 around the filamentous plaques in AsymAD MFGs than in AD MFGs (Fig. 3c), whereas GFAP levels were decreased in intermediate plaques (Fig. 3f). No significant changes were observed in IBA1 coverage around intermediate or compact plaques; likewise, no significant changes were detected in GFAP coverage around filamentous or compact plaques. Taken together, our observations indicate that there is a difference in the distribution of IBA1 associated with amyloid plaques between AsymAD and AD cases, with greater microglial abundance in the vicinity of filamentous amyloid plaques in AsymAD MFGs. These data suggest the presence of a protective niche in the proximity of plaques in the MFG of AsymAD cases that could prevent pathological tau conversion despite the presence of Aβ plaques.

### The genetic landscape of the amyloid plaque microenvironment in AsymAD cases involves increased autophagy-, endocytosis- and phagocytosis-related pathways

**Fig. 4:**
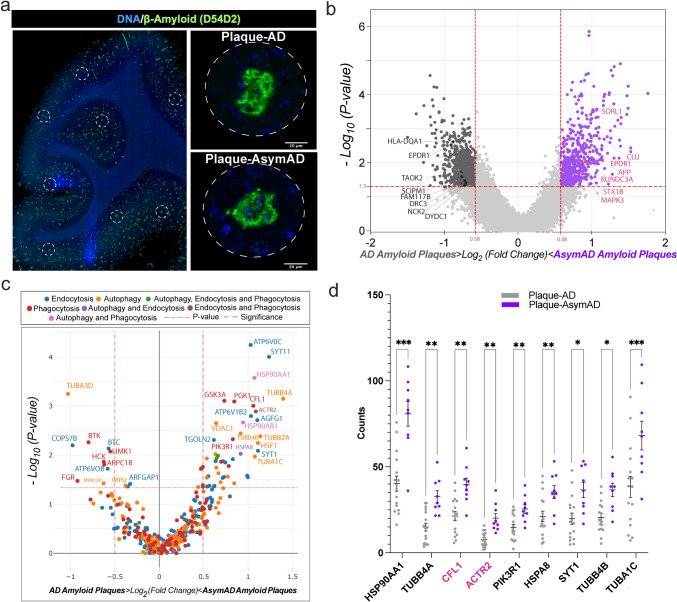
Characterization of the AsymAD and AD plaque niches using spatial whole transcriptomics. **a** After staining for Aβ-amyloid (green) and DNA (blue), regions of interest (ROIs) containing Aβ-amyloid plaques in the AD and AsymAD MFGs were selected; Sixteen ROIs from two AD cases and nine ROIs from one AsymAD case. **b** Volcano plot of the DEGs found in AD amyloid plaques *versus* AsymAD amyloid plaque ROIs. **c** Volcano plot of AsymAD versus AD, highlighting genes from the endocytic- autophagy- and phagocytic- pathways. In **b** and **c**, vertical dashed lines indicate a fold change over 1.5 (log_2_ FC =  ± 0.58), and horizontal dashed lines indicate a *p* value of 0.05 (− log_10_ *p* value = 1.3). *p* value < 0.05 adjusted for multiple analyses using the Benjamini–Hochberg procedure with a false discovery rate (FDR) of 0.01. **d** Normalized counts of some of the genes highlighted in c. Data are shown as the mean ± SEM, 2-way ANOVA, following Šídák’s multiple comparisons test; one dot represents one ROI; *n* = 9–16 per case (*p* value ***; < 0.0001, **; < 0.0001 and *; < 0.05)

To understand the differences observed in the amyloid plaque microenvironment of AsymAD compared to AD MFGs, we performed an explorative GeoMx human WTA to measure over 18,000 protein-coding genes cross-referenced with the HUGO and NCBI RefSeq databases. We selected MFG sections from AsymAD and AD brains and analyzed 16–19 ROIs per sample. Each ROI encompassed a ratio of 30–50 μm from the Aβ plaque core and its immediate microenvironment in the gray matter (Fig. 4a). We identified a total of 1799 DEGs. Among these genes, 813 were upregulated in the Aβ plaque microenvironment of AsymAD (plaque-AsymAD), while 986 were upregulated in AD (plaque-AD) (Fig. 4b and Supplementary Excel 1). Interestingly, within the upregulated genes found in the plaque-AsymAD microenvironment, there were seven DEGs also present in a genome-wide list of significant variants associated with AD and related dementias [6] (Highlighted in Fig. 4b and Supplementary Excel 1—GWAS and AD hit comparisons). Furthermore, 48 DEGs in plaque-AsymAD and 9 DEGs in plaque-AD were previously linked to AD targets through RNA-seq, proteomic, and metabolomic studies. These findings were compiled from a list of nominated targets stemming from the Accelerating Medicines Partnership in AD (AMP–AD) consortium and the broader AD research community (AGORA) [109]. Using the 1799 DEGs, we performed a pathway enrichment analysis using the GeoMx DSP analysis suite. Interestingly, pathways related with endocytosis (clathrin-mediated endocytosis, cargo recognition for clathrin-mediated endocytosis, transferrin endocytosis and recycling), autophagy (autophagy, macroautophagy, selective autophagy, chaperone mediated autophagy) and phagocytosis (regulation of actin dynamics for phagocytic cup formation, response of Mtb to phagocytosis, Fcgamma receptor (FCGR) dependent phagocytosis) were upregulated more than four times in the amyloid plaque microenvironment of plaque-AsymAD MFGs when compare with plaque-AD MFGs (Bold pathways in Supplementary Excel 2). From the 813 upregulated genes in the plaque-AsymAD ROIs, 57 DEGs were in the endocytic-, autophagy- and phagocytic-related pathways. In contrast, only 12 DEGs out of the 986 upregulated genes in plaque-AD ROIs belonged to these pathways (Fig. 4c and Supplementary Excel 2). These findings align with a recent study in which elevated gene expression related to the early stages of autophagy was demonstrated to be responsible for maintaining synaptic integrity through the efficient removal of tau oligomers in the hippocampus of AsymAD subjects [98]. Furthermore, genetic variants linked to autophagy processes may contribute to resistance against amyloid plaques and NFTs in centenarians [107] and previous evidence suggest that phagocytosis and endocytosis might underlie synaptic resilience in AsymAD individuals [92, 98]. These mechanisms has been shown to be impaired in AD brains [45, 55].

We further evaluated the expression of the DEGs upregulated in the plaque-AsymAD microenvironment by plotting the normalized counts in plaque-AsymAD versus plaque-AD ROIs. We observed a consistent and significant upregulation of nine DEGs in plaque-AsymAD versus plaque-AD ROIs (Fig. 4d).

### AsymAD microglia exhibit increase levels of actin-based cell motility proteins ARP2 and CAP1 within the amyloid plaque microenvironment

Although our data support previous evidence suggesting that endocytosis and phagocytosis might underlie synaptic resilience in AsymAD individuals [27, 92], the literature also indicates the role of some of these DEGs in actin-based motility (pink highlighted genes in Fig. 4d). Interestingly, a recent study that used mass spectrometry-based proteomics has pointed out the crucial role of actin network proteins in neurons to confer resilience to AD [39]. The ACTR2 (encoding ARP2) and CFL1 (Cofilin 1) genes play significant roles in actin remodeling, enabling both baseline movement (ruffling or branching) and chemotactic motility (migration) [18, 28, 29]. While ARP2 (Actin related protein 2) is part of the Arp2/3 complex, and its role is to engage with actin monomers to start the formation of a new filament branch [75], CFL1, depolymerizes actin filaments to make them available for the formation of new actin structures. While the study of these genes has been focused on their role in regulating spine morphology to preserve synapses [51, 74, 99], the literature also highlights their crucial involvement in microglial motility [18, 20]. Indeed, in human brains, CFL1 is expressed across various cell types, including astrocytes, neurons, and microglia, while ACTR2 is predominately expressed in microglia and macrophages [108]. Given that different microglial phenotypes, characterized by distinct gene signatures, have been identified in response to Aβ amyloid in both human and AD mouse models [47, 59, 69, 88], we also checked the expression levels of CFL1 and ACTR2 using these databases. None of the aforementioned genes were exclusively associated with one specific microglial phenotype. Furthermore, we compared our GeoMx WTA DEGs with gene signatures linked to disease-associated microglia (DAMs) in humans and mice (also known as activated response microglia—ARM—or neurodegenerative microglia -MGnD-), identifying several genes associated with this profile in both, AD (65 DEGs) and AsymAD (306 DEGs) plaque microenvironments (Supplementary Excel 1—Microglia clusters).

Considering the spatial transcriptomics data, the prevailing expression of ACTR2 in microglia, and the emerging role of ACTR2 in microglial motility, we aimed to investigate whether its protein levels were altered in the microenvironment of AsymAD plaques by analyzing a larger number of cases. Additionally, to determine if other components of actin-based motility processes, absent in the previous pathways, are altered in the plaque-AsymAD microenvironment, we assessed the levels of CAP1. CAP1 plays a role in facilitating the release of CFL1 from ADP-actin filaments [29].

**Fig. 5:**
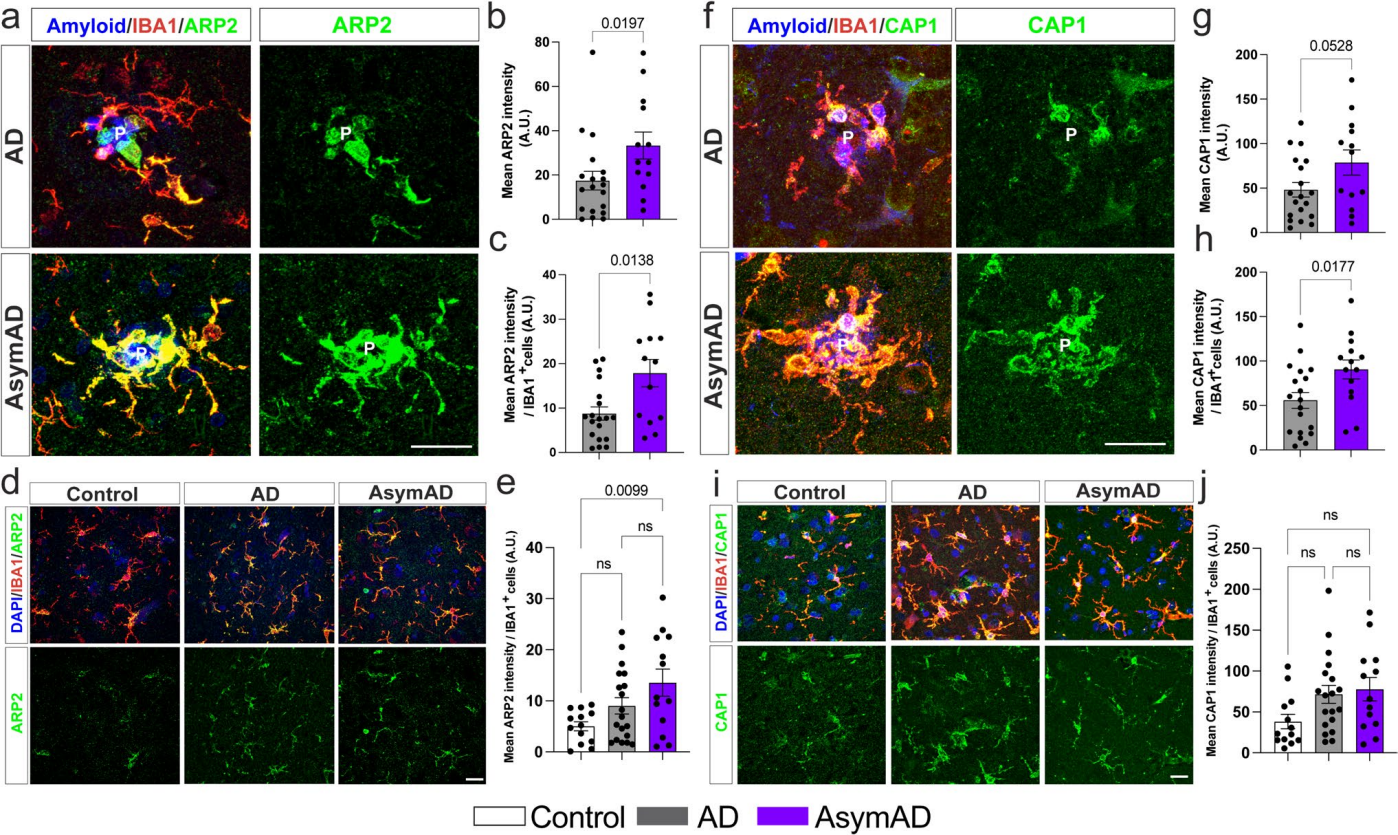
Microglial ARP2 and CAP1 levels are increased in the plaque microenvironment of AsymAD MFGs. ARP2 (green) (**a**) or CAP1 (green) (**f**) staining in the brain of AsymAD and AD cases. IBA1 (red) and NucBlue (blue) were used to identify microglia and cell nuclei/amyloid, respectively (P = Plaque Location). Green images indicate ARP2 (**a**)- and CAP1 (**a**, **f**)-positive staining. **b**, **g**. Quantification of overall ARP2 (b) or CAP1 (g) intensity within 50 μm of the core plaque*.* **c**, **h** Mean ARP2 (c) or CAP1 (h) intensity within the IBA1^+^ area. **d**, **i** ARP2 (d) or CAP1 (**i**) and IBA1 immunostaining in plaque-free areas **e**, **j**. Quantification of the mean ARP2 (**e**) or CAP1 (**j**) intensity per IBA1 cell in plaque-free areas. In b, c, g and h, *n* = 14–19 cases per group were analyzed. The data are shown as the mean ± SEM, and significance was determined by unpaired Student’s *t* test. In e and j, *n* = 13–19 cases per group were analyzed. The data are shown as the means ± SEMs; one-way ANOVA was used following Tukey’s multiple comparisons test

First, using antibodies against ARP2 or CAP1(green) and IBA1 (red), we evaluated the levels and distribution of these proteins in the proximity of amyloid plaques (blue) inside and outside microglia (Fig. 5a, d, f, i). Analysis of the overall fluorescence intensity of ARP2 revealed a significant increase in its expression in plaque-AsymAD versus that in plaque-AD (Fig. 5b). Furthermore, the mean of ARP2 immunoreactivity increased when evaluating only the IBA1-positive (IBA1^+^) area of the AsymAD plaque microenvironment. This indicates elevated ARP2 levels in microglia surrounding the amyloid plaques in AsymAD MFGs compared to AD MFGs (Fig. 5c). We also analyzed ARP2 immunoreactivity in plaque-free areas (Fig. 5d). Although we did not find differences in the ARP2 intensity per IBA1 cell between AsymAD and AD cases, AsymAD MFGs showed significantly higher levels of ARP2 than did control brains (Fig. 5e), suggesting that AsymAD cases may possess higher baseline levels of ARP2. Similar to these findings, we observed an increase in the CAP1 levels within the amyloid plaque microenvironment in AsymAD cases (Fig. 5f–h). This increase in CAP1 levels was not statistically significant between conditions when overall intensity was determined (Fig. 5g); however, a significant increase in CAP1 levels was observed within microglia (Fig. 5h). No differences between conditions were found in plaque-free areas (Fig. 5j). Given the increased microglial ARP2 levels in AsymAD compared to AD and controls cases, and its key role in generating new microglial ramifications and enhancing surveillance [20], we sought to assess the role of ARP2 in microglia direct motility. To do so, we manipulated the ACTR2 gene in the HMC3 human microglial cell line and conducted a scratch-wound assay (Supplementary Fig. 6a, b). The overexpression of ACTR2 resulted in enhanced microglial occupancy within the scratch area, leading to a significant reduction in the wound area compared to that observed in the control by the end of the assay. Conversely, the downregulation of ACTR2 maintained a consistently unaltered wound area from 40 h until the conclusion of the assay (Supplementary Fig. 6c).

Overall, these data suggest that in AsymAD cases, microglia exhibit more efficient actin-based motility mechanisms, which could heighten actin dynamics, facilitating efficient migration toward the vicinity of the plaque, promoting the elongation of microglial branches, and enhancing engagement with the plaque.

## Discussion

Our study explored the molecular mechanisms within the microenvironment of amyloid plaques in AsymAD individuals, which may contribute to synapse preservation. These mechanisms were linked to a distinct cell response comprising increased expression of autophagy-related genes, and actin-based cell motility-related genes, such as ARP2 and CAP1. These genes potentially facilitate the deposition of Aβ into dense-core plaques and prevent amyloid-driven tau pathogenesis within the amyloid plaque microenvironment of filamentous plaques.

Substantial evidence suggests that microglia around plaques are key regulators of their morphology in AD mouse models. Initial observations revealed that reactive microglia encircle amyloid plaques and sequester Aβ amyloid within their cytoplasm in vitro [48, 104]. Subsequent studies supported these observations; in the CRND8 and 5xFAD AD mouse models, microglia have been shown to form a tight barrier around plaques, preventing their growth. Moreover, in regions lacking microglial processes, neuritic dystrophy is more severe, and these protective mechanisms are reduced during aging [15]. Casali et al. showed that pharmacological depletion of microglia in ten-month-old 5xFAD mice reduced the plaque burden, and the remaining plaques exhibited increased diffuse-like plaques and fewer compact-like shapes, together with increased dystrophic neuritis [12]. Similarly, Spangenberg et al. observed a reduction in dense-core plaques in the cortex of the same mouse model following chronic administration of an inhibitor of microglial proliferation [84]. Using the APP/PS1 AD mouse model, Huang et al. showed that genetic ablation of tyrosine kinase TAM receptors inhibited microglial phagocytosis and decreased dense-core plaque density in the cortex and hippocampus after 12 months of age; these changes were not due to any changes in the production of Aβ peptides [38]. In another study, exposure to ozone decreased the association of phagocytic microglia with amyloid plaques, impairing their ability to form a protective barrier and exacerbating dystrophic neuritis [33]. These findings suggest that microglia are potentially critical regulators of plaque conformation and that dense-core Aβ plaques do not form spontaneously but are constructed from loosely organized Aβ material produced by phagocytic microglia. Moreover, the importance of microglial plaque association lies in the ability of microglia to prevent amyloid plaques from causing synaptic damage. The variability in microglia and astrocyte distribution according to the plaque phenotype in AsymAD versus AD brains provides insight into the complexity of the amyloid-associated glial response. One study showed that reactive astrocytes and activated microglia respond differently to Aβ plaque formation; while microglia respond directly to the presence of plaques, astrocytes are associated with neuritic damage that occurs when synapses are already dysfunctional [81]. Therefore, a stronger microglial barrier surrounding plaques in the cortices of AsymAD cases may protect against synaptic derangement and neuritic damage, which, in turn, could mitigate the astrocytic response.

Studies in AD mouse models support the importance of the Aβ plaque microenvironment in promoting the pathological conversion of tau. In a mouse model of amyloidosis, it has been observed that Aβ plaques create a unique environment that triggers tau phosphorylation within dystrophic neuritis [53]. Furthermore, He et al. reported that when human AD-derived tau was injected into a plaque-bearing 5xFAD mouse model, Aβ plaques facilitated the conversion and seeding of pathological tau in dystrophic neurites during the early stages of pathology. This tau propagation could occur through axonal transmission to the neuronal soma and dendrites, ultimately leading to NFT formation [34]. However, NFT formation and synaptic deterioration are not entirely dependent on Aβ plaque–mediated tau pathogenesis [34], which could explain why AsymAD subjects still exhibit a considerable quantity of NFTs independent of NP-tau. Consistent with our findings, previous studies have shown that AsymAD cases exhibit an overall deficiency in oligomeric and soluble phospho-tau species, indicating fewer pathological tau species in these cases than in AD cases [8, 73, 83]. Moreover, a recent study demonstrated that in addition to NFT deposition, there is a notable increase in tau oligomer-containing synapses within microglia and astrocytes during the early stages of AD but not in AsymAD cases [89]. Interestingly, it has been reported that neuronal degeneration in AD could occur via non-NFT formation in multiple neurons [44]. Given the findings of the studies mentioned above, neurotoxic tau oligomers may be responsible for a secondary pathway of neuronal death independent of NFT formation. Therefore, the evidence provided in the current study suggests that the plaque microenvironment in AsymAD individuals does not facilitate the formation of soluble tau species with strong seeding activity, unlike in neurotoxic tau oligomers formation in AD brains [60].

It is uncertain whether microglia provide beneficial but insufficient support during disease progression or whether they are effective in early disease stages but lose effectiveness and even become harmful later. Extensive evidence shows that autophagy and microglial phagocytosis are impaired in AD and aged brains [24, 30, 94, 103]. However, it has been reported that genetic variants of genes related to autophagy functions are involved in resilience against AD neuropathology [82, 92, 107]. How these processes contribute to AD resilience remains an area of active investigation, and extensive exploration has not yet been conducted. Studies in AD mouse models support the idea that more efficient microglia confer protection against amyloid plaque toxicity through their phagocytic activity. In Trem2 or Dap12 haplodeficient mice and humans carrying the R47H mutation in the TREM2 gene, the microglia demonstrate a markedly reduced ability to envelop amyloid deposits, decreasing compact plaque phenotypes and increasing amyloid fibril exposure to adjacent neurites, which is associated with tau hyperphosphorylation [102, 106]. Moreover, a recent study revealed a high abundance of activated microglia in the cortices of an NDAN patient cohort. The authors also reported higher levels of the microglial phagocytic complex TREM2/DAP12 in Aβ amyloid plaques in NDAN than in those in AD, concluding that microglia surrounding Aβ plaques in NDAN are hyperactive and more effectively recognize damaged synapses with greater phagocytic capacity than microglia in AD [27]. Interestingly, proteomics analyses of neocortex tissues from pathologically diagnosed preclinical AD cases revealed an increase in processes associated with vesicle endocytosis and the secretory pathway [54]. Although these data are not cell specific, they suggest that these processes are efficient in the early stages of pathology. Our GeoMx WTA revealed several genes associated with cell engulfment (endocytosis and phagocytosis) and autophagy within the plaque microenvironment of AsymAD MFGs, similar to the findings in early stages of the pathology in mouse models and preclinical AD cases. Therefore, microglia in AsymAD cases may maintain their ability to cleave Aβ amyloid even years after plaque formation.

Microglial function is highly dependent on baseline motility, which consists of the extension, retraction, and movement of microglial processes [37, 70], allowing membrane ruffling. It has been shown that aging reduces microglial adhesion and migration toward fibrillar Aβ in APP/PS1 mice and even in wild-type mice [23]. At the molecular level, cytoskeleton reorganization is necessary to carry out these processes [29]. In this context, ARP2, CAP1, and CFL1 are proteins related to engulfment processes through actin-based motility mechanisms [28, 29], that were recently associated with AD resilience according to protein coexpression analysis [39]. In the context of AD, an overall reduction in ARP2 has been found in human AD parietal cortex tissue [51]. Although there is no clear evidence of CFL1 and CAP1 changes in AD [28, 72], CFL1 is one of the main components of actin/cofilin rods, which are insoluble aggregates that lead to neurodysfunction [4, 101]. Interestingly, we found that microglia in plaque-free areas exhibited significantly greater levels of ARP2 in AsymAD MFGs than those observed in healthy controls. This finding led us to speculate that AsymAD cases may have a higher basal level of microglial ARP2 and, consequently, enhanced motility, as evidenced by increased microglial motility observed in vitro, potentially occurring even before plaque formation. Furthermore, AsymAD brains may possess the ability to increase ARP2 expression in response to amyloid pathology. Considering a previous study in a transgenic mouse model of AD demonstrating that efficient microglial clustering around Aβ plaques mitigates amyloid-driven tau seeding [32], AsymAD individuals might be characterized by the presence of microglia with enhanced migration and extension toward newly formed amyloid plaques. This ability of microglia to efficiently cluster around plaques could reduce the effect of amyloid in promoting tau seeding and subsequently mitigate synaptic deterioration. It is worth to mention that ARP2 is not expressed solely in microglia; therefore, we cannot rule out effects from other cell types [35, 85]. Our GeoMx WTA also showed enrichment of synaptic and neurotransmission release-related genes (VAMP2, SYT1 and SYT11) and genes encoding microtubule proteins within the axons (TUBA1C and TUBB4A) in plaque-AsymAD versus plaque-AD. It is well established that the absence of dementia in AsymAD individuals is partly due to synaptic preservation and, hence, neuron survival [31, 67].

Finally, this study proposes a potential mechanism by which AsymAD cases may resist or delay the pathological processes contributing to the formation of synaptotoxic soluble tau species with seeding activity within the amyloid plaque niche. This finding highlights the significance of an efficient microglial response to amyloid plaques. While our findings reveal new insights into the resilience to AD pathology involving microglial mechanisms, further studies with larger sample sizes are needed to assess sex differences. Furthermore, our findings are limited to the middle frontal gyrus brain area, and this limitation should be considered when interpreting the data. Finally, mechanistic experiments to assess the specific response of microglial ARP2/CAP1 to Aβ amyloid and tau will be necessary to explore their role in synaptic protection and contribution to AD resilience.

## Conclusion

Our findings reveal a novel mechanism by which AsymAD cases can maintain normal cognition and achieve resilience against AD. We observed that microglia in AsymAD MFGs exhibit increased expression of proteins involved in a more efficient chemotactic motility than those in AD brains. These could allow microglia to remodel their branches and surround the amyloid plaque, facilitating the engulfment and clearance of toxic Aβ aggregates, which may mitigate Aβ-associated tau pathogenesis and decrease tau seeding. Our discoveries have important implications for the development of interventions to halt synaptic damage in AD patients and forestall subsequent cognitive impairments and dementia.

### Supplementary Information

Below is the link to the electronic supplementary material.

Supplementary file1 (PDF 149135 KB)

Supplementary file2 (XLSX 2013 KB)

Supplementary file3 (XLSX 406 KB)

## Data Availability

The data that support the findings of this study are openly available in Synapse.org at 10.7303/syn61908908 and upon request.
