# Supplementary material for: Enhanced microglial dynamics and a paucity of tau seeding in the amyloid plaque microenvironment contribute to cognitive resilience in Alzheimer’s disease
Source: Acta Neuropathol. 2024 Aug 5;148(1):15. doi: 10.1007/s00401-024-02775-1 (PMC11300572; doi:10.1007/s00401-024-02775-1)
Supplement: Supplementary file 1 — Supplementary file1 (PDF 149135 KB) [file 401_2024_2775_MOESM1_ESM.pdf]

SUPPLEMENTARY FIGURES

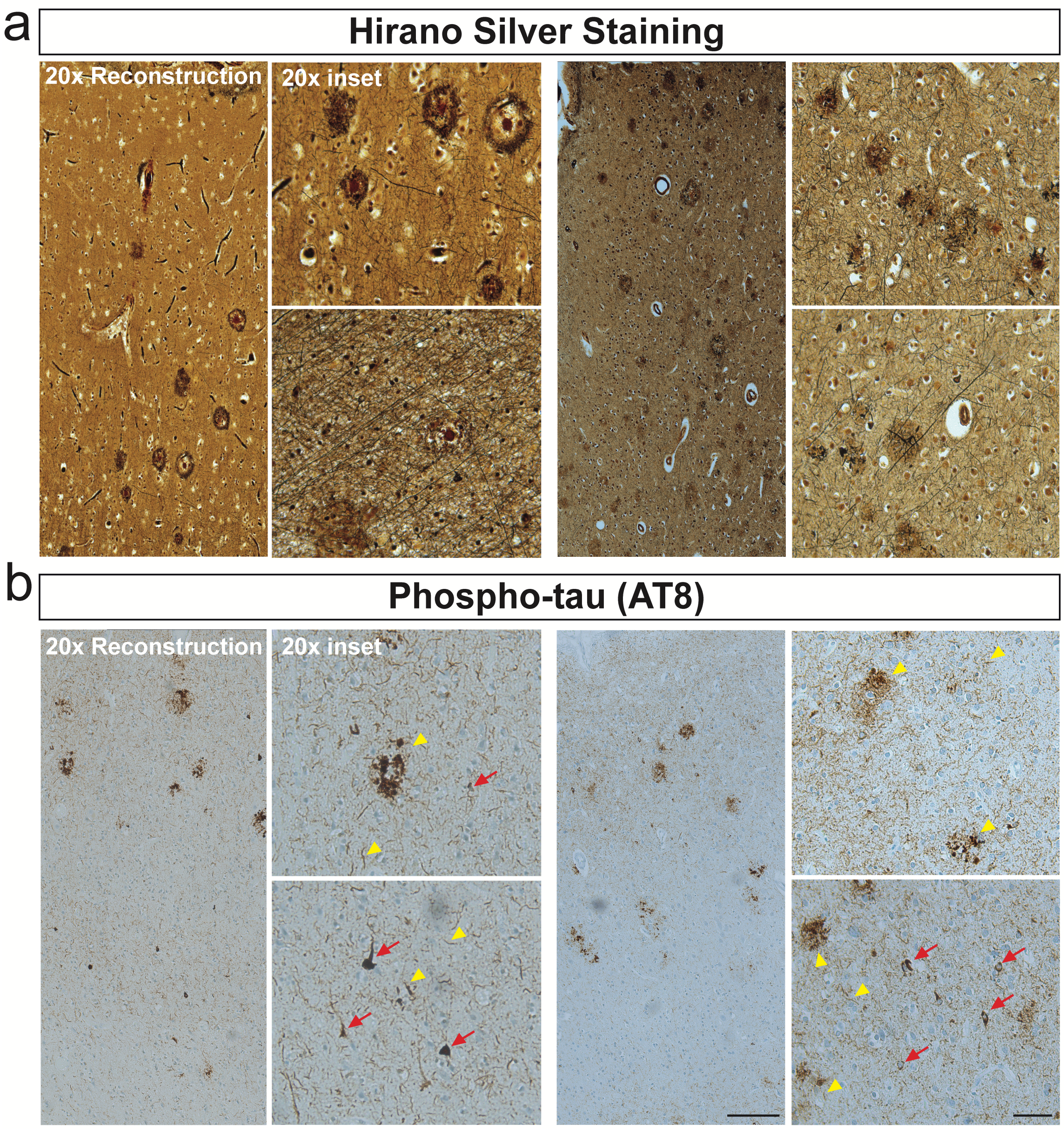

Supplementary Figure 1: Alzheimer's disease pathology in the visual cortex of AsymAD cases. a-b. Representative images of Hirano silver staining (a) and AT8 immunostaining (b) in the visual cortex of AsymAD cases (Cases 41 and 42 in Table I). Both stainings revealed plaques, neuritic plaques and neuritic tau (yellow arrowheads), and NFTs (red arrows), along with a dense network of AT8-positive neurites in the background. The presence of AD lesions in the visual cortex was used to classify AsymAD cases with Braak stages V/VI, as described in Table I. Scale bar: 100 µm (20x reconstruction) and 40 µm (insets).

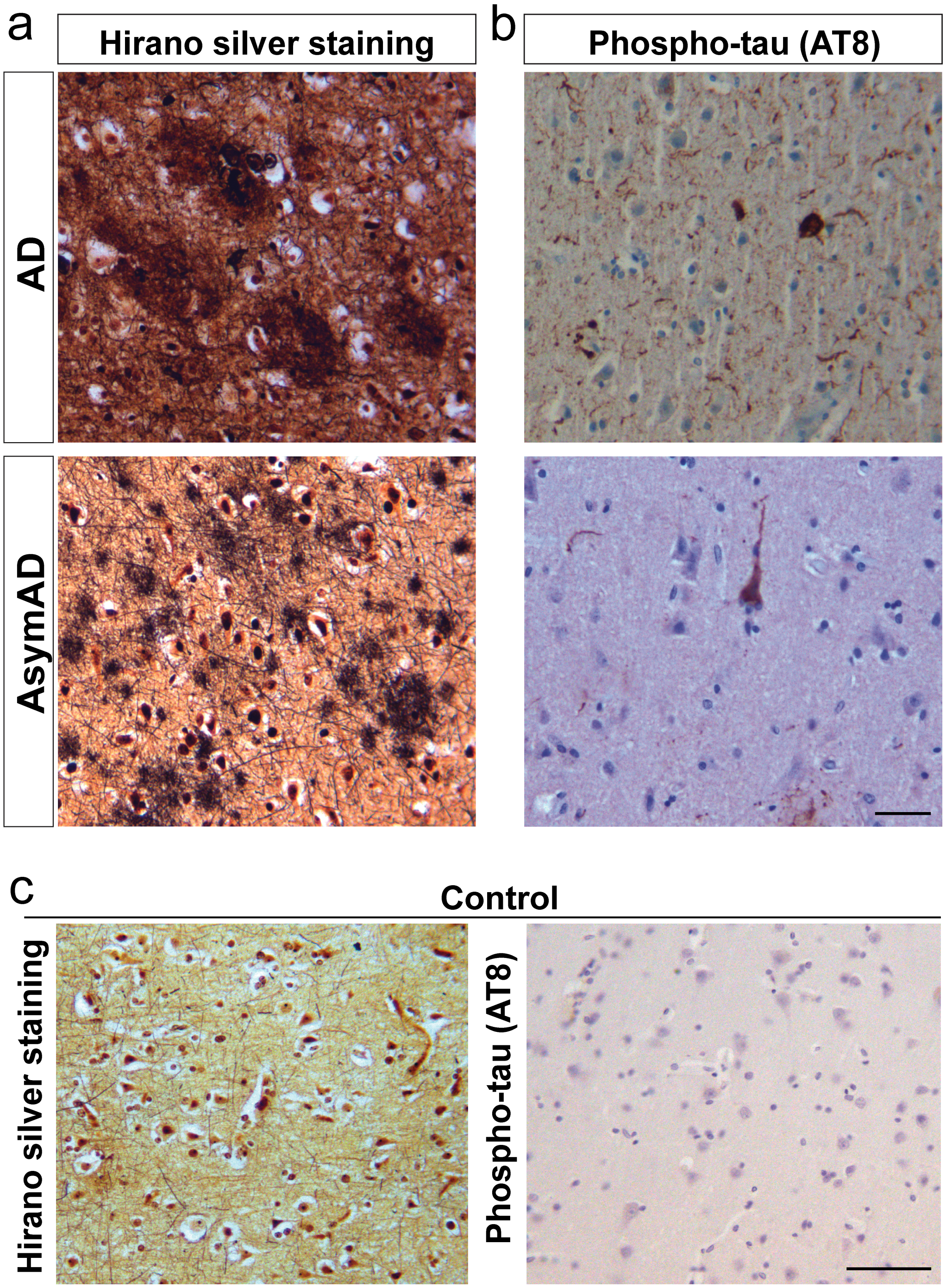

Supplementary Figure 2: Middle frontal gyrus autopsies in AsymAD cases with substantial AD pathology. a-b. Histopathology of AD and AsymAD cases confirmed the presence of amyloid plaques by Hirano silver staining (a) and neurofibrillary tangles (NFTs) by AT8 (b), an antibody that recognizes the pathological phospho-sites at Ser202/Thr205 of the tau protein. Scale bar: 20 μm. **c.** No reactivity for any of these pathological markers was found in age-matched control brains. Scale bar: 50 μm.

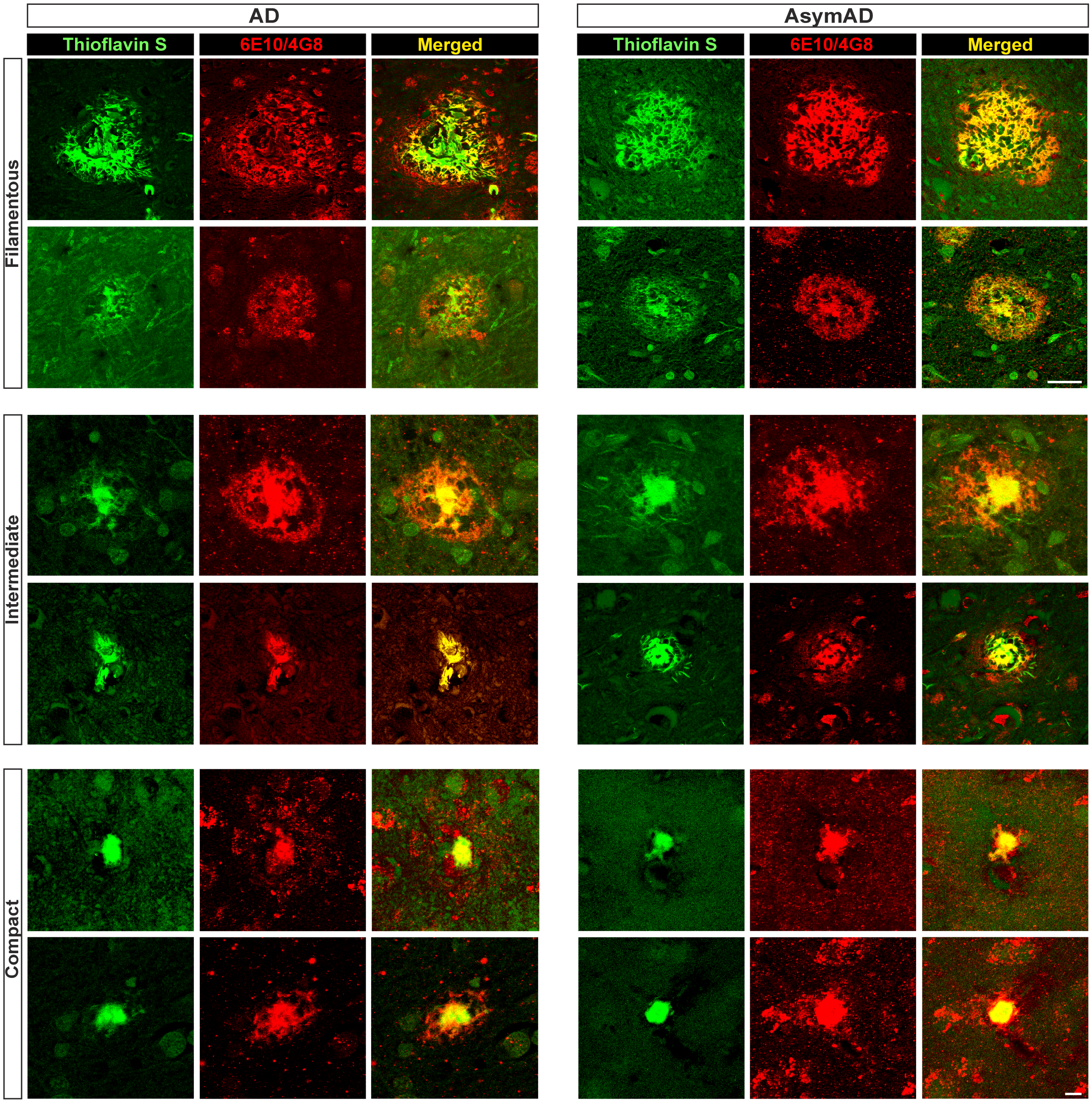

Supplementary Figure 3: Confocal immunofluorescence of the three thioflavin S plaque subtypes detected by 6E10 and 4G8 antibodies**.** Scale bar: 30 µm and 5 µm.

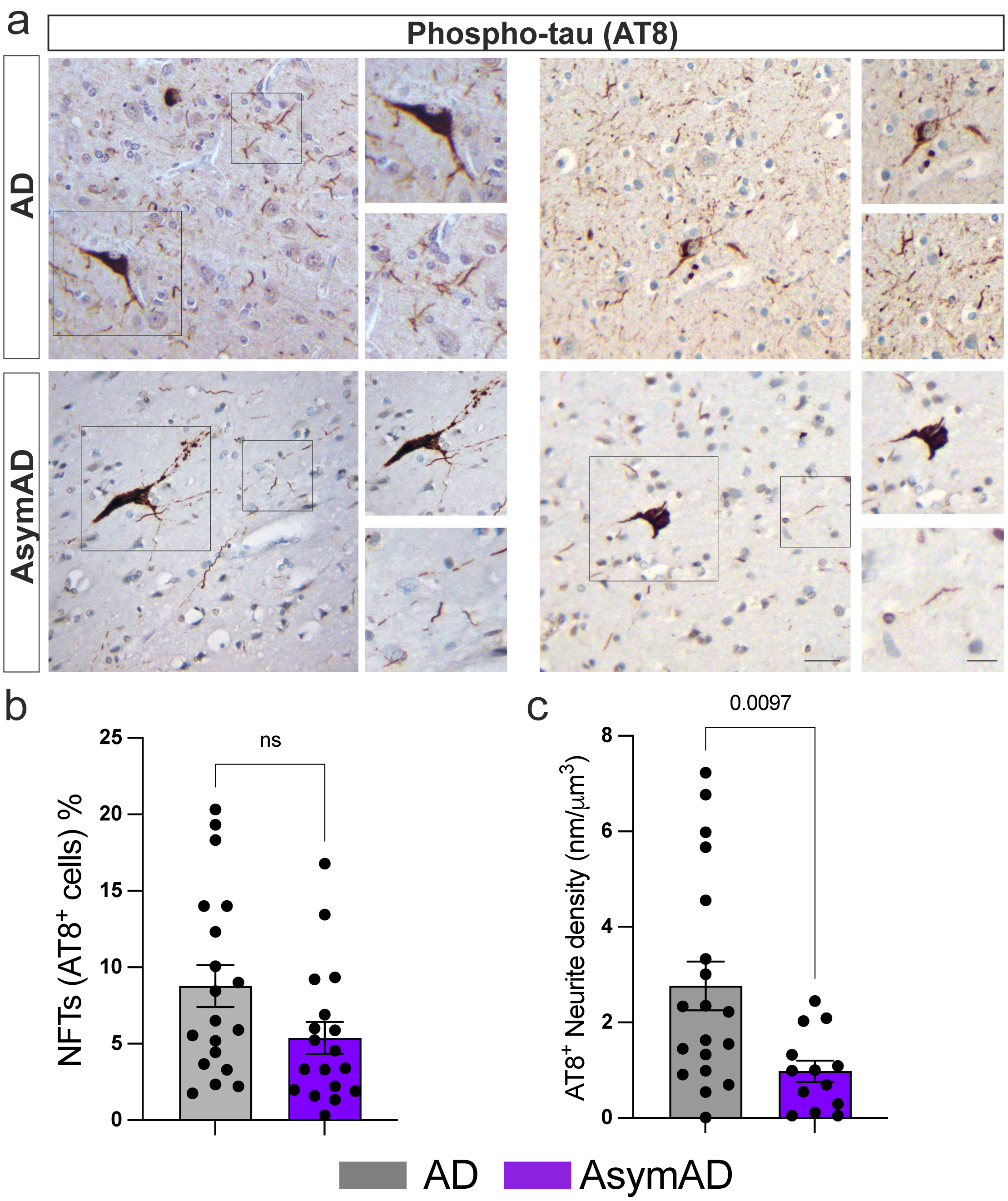

Supplementary Figure 4: AsymAD MFGs show a similar number of NFTs to AD brains but reduced AT8-positive neuritic staining. a. Phospho-tau staining in AD and AsymAD MFGs**.** Both representative figures correspond to cases classified as CERAD C and a NFTs Braak stage of VI. Scale bar: 20 µm and 10 µm (insets) **b.** Percentage of NFTs (AT8-positive) **c.** Quantification of AT8-positive neurites. The data are shown as the means ± S.E.M.s; unpaired Student's *t* test; *n = 13–19* cases per group.

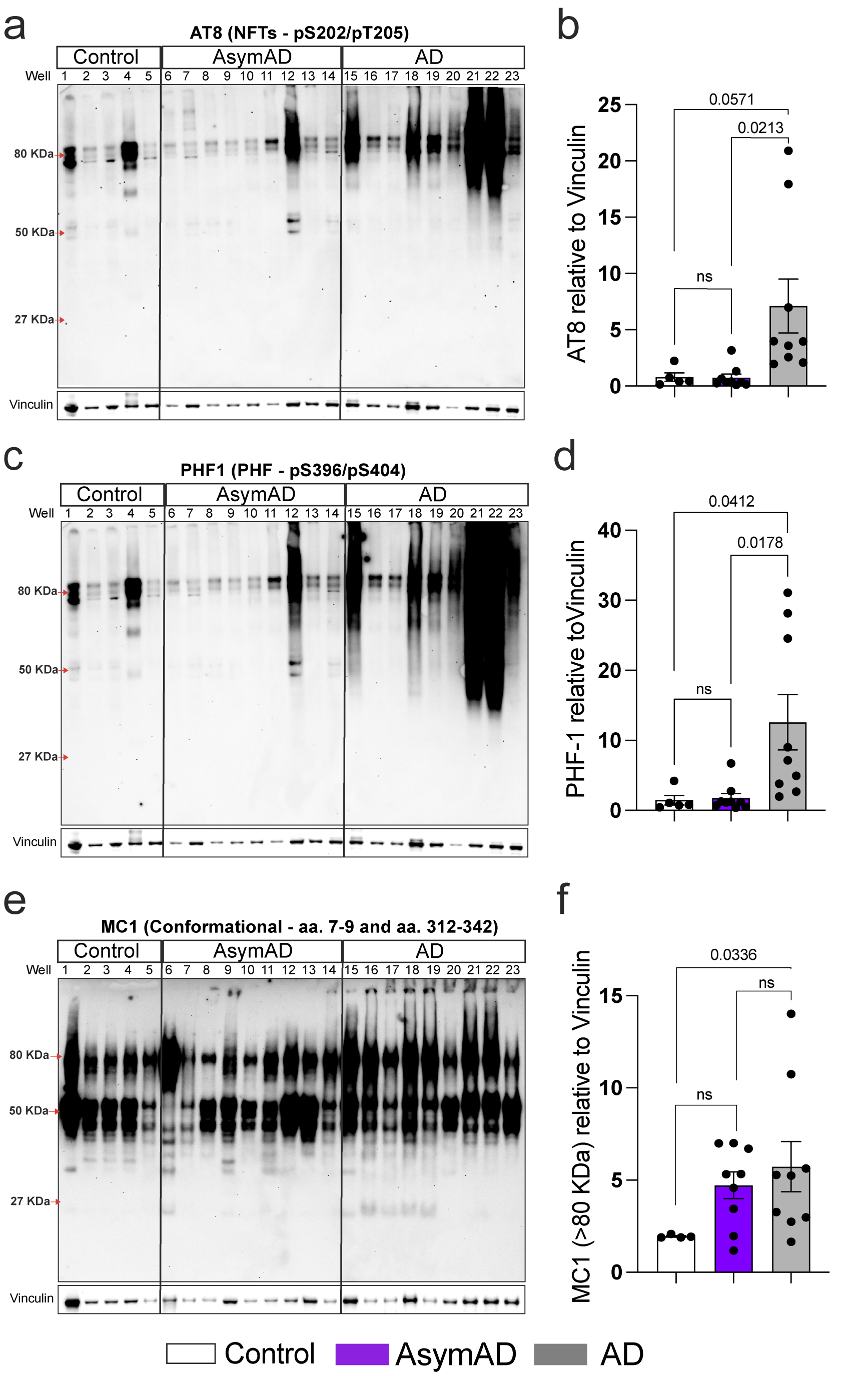

Supplementary Figure 5: Tau species in total soluble extracts of AD, AsymAD, and aged-matched control brains. a-b. Western blot of PHF1 (pTauS396/S404) (a), AT8 (pTauS202/Thr205) (c), and MC1 (e) and their corresponding quantifications (b, d, and f). The data are shown as the mean ± S.E.M. Significance was determined by one-way ANOVA, and the experiments were performed with $n = 5–9$ cases per group.

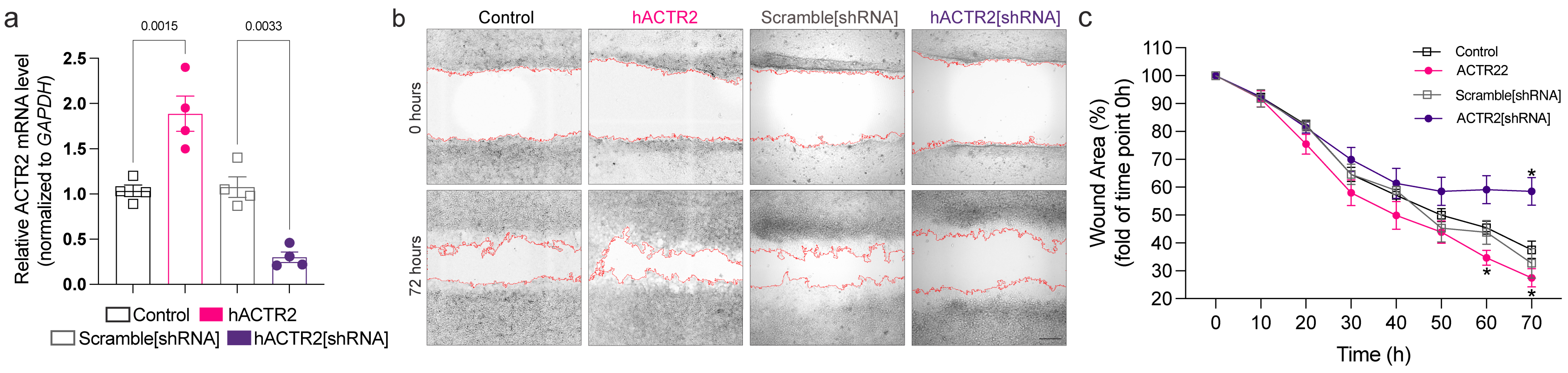

Supplementary Figure 6: Modulation of the ACTR2 gene alters microglial migration *in vitro*. HMC3 microglia were transfected with vectors to overexpress (hACTR2) and downregulate (ACTR2[shRNA]) the ACTR2 gene **a.** Three days post-transfection, the overexpression of ACTR2 increased the ACTR2 mRNA level by 44%, whereas ACTR2 shRNA decreased the ACTR2 mRNA level by 71% in HMC3 cells ($n = 4$ per group) **b.** A scratch was made in the middle of the wells with the IncuCyte® 96-well scratch wound maker tool. The red delimitations indicate the scratch area recorded at 0 and 72 h. **c.** Quantitative analysis of wound closure as a function of time. The data are shown as the means ± S.E.M.s*;* one-way ANOVA and Tukey's multiple comparisons test (*p* value *;<0.05).
